# Hyper-IgG4 disease: report and characterisation of a new disease

**DOI:** 10.1186/1741-7015-4-23

**Published:** 2006-10-06

**Authors:** Guy H Neild, Manuel Rodriguez-Justo, Catherine Wall, John O Connolly

**Affiliations:** 1 UCL Centre for Nephrology, Royal Free Hospital, London NW3 2QG, UK; 2 Institute of Urology and Nephrology, Middlesex Hospital, London W1T 3AA, UK; 3 Department of Histopathology, Royal Free and University College Medical School, University College Hospital, Rockefeller Building, London WC1E 6JJ, UK

## Abstract

**Background:**

We highlight a chronic inflammatory disease we call 'hyper-IgG4 disease', which has many synonyms depending on the organ involved, the country of origin and the year of the report. It is characterized histologically by a lymphoplasmacytic inflammation with IgG4-positive cells and exuberant fibrosis, which leaves dense fibrosis on resolution. A typical example is idiopathic retroperitoneal fibrosis, but the initial report in 2001 was of sclerosing pancreatitis.

**Methods:**

We report an index case with fever and severe systemic disease. We have also reviewed the histology of 11 further patients with idiopathic retroperitoneal fibrosis for evidence of IgG4-expressing plasma cells, and examined a wide range of other inflammatory conditions and fibrotic diseases as organ-specific controls. We have reviewed the published literature for disease associations with idiopathic, systemic fibrosing conditions and the synonyms: pseudotumour, myofibroblastic tumour, plasma cell granuloma, systemic fibrosis, xanthofibrogranulomatosis, and multifocal fibrosclerosis.

**Results:**

Histology from all 12 patients showed, to varying degrees, fibrosis, intense inflammatory cell infiltration with lymphocytes, plasma cells, scattered neutrophils, and sometimes eosinophilic aggregates, with venulitis and obliterative arteritis. The majority of lymphocytes were T cells that expressed CD8 and CD4, with scattered B-cell-rich small lymphoid follicles. In all cases, there was a significant increase in IgG4-positive plasma cells compared with controls. In two cases, biopsies before and after steroid treatment were available, and only scattered plasma cells were seen after treatment, none of them expressing IgG4. Review of the literature shows that although pathology commonly appears confined to one organ, patients can have systemic symptoms and fever. In the active period, there is an acute phase response with a high serum concentration of IgG, and during this phase, there is a rapid clinical response to glucocorticoid steroid treatment.

**Conclusion:**

We believe that hyper-IgG4 disease is an important condition to recognise, as the diagnosis can be readily verified and the outcome with treatment is very good.

## Background

While investigating a patient who had an uncharacterised multisystem disease with evidence of a severe acute-phase response, we found that similar rare cases had been described, usually as single reports [1], under an alarming list of synonyms [2] (Table 1). These conditions are all characterised by chronic inflammation leading to dense fibrosis, and retroperitoneal fibrosis (RPF) is a typical example. The principal synonyms that we found for these fibrosing conditions are: pseudotumour, myofibroblastic tumour, plasma cell granuloma, systemic fibrosis, xanthofibrogranulomatosis, and multifocal fibrosclerosis (Table 1). As can be seen in Tables 1 and 2, these conditions, such as RPF, Reidel's thyroiditis and sclerosing pancreatitis, are usually localised to one or two organs but can present with systemic multisystem disease.

**Table 1:** Systemic fibrosis synonyms

Pseudotumour, inflammatory pseudotumour, fibrous pseudotumour	Systemic[83-85]	Lung[86-89], liver[87,90-93], breast[94], pancreas[83,95-99], orbit[100], RPF[98-101], mesentery[102]
Inflammatory myofibroblastic tumour, myofibroblastoma	Systemic	Pancreas[96], lung[46,103,104], brain[105], breast[106]

Plasma cell granuloma	Systemic	lung[107,108], brain[107]

Systemic fibrosis, generalized form of Ormond's disease, systemic idiopathic fibrosis, idiopathic systemic sclerosing disease	Systemic[1,109-111]	PRF[109,111-113], orbit[112], lung[114,115]

Xanthofibrogranulomatosis, xanthogranuloma	Systemic[1,2,116,117]	RPF[101,118-121], brain[118,122], orbit[123]

Multifocal fibrosclerosis, multifocal idiopathic fibrosclerosis	Systemic[84,85,124-128]	RPF[124,125,128-133], orbit[124,129-131,134] thyroid[127,128,132,133], pancreas[9]

RPF, retroperitoneal fibrosis.

**Table 2:** Conditions associated with systemic fibrosis

Name	Synonyms	
RPF	Ormond's disease	Systemic[109,111,113,135], lung[32,32,53,115,136,136,137], liver[138], breast, pancreas[5,139-141],

Retro-orbital tumour	Fibrous pseudotumour of the orbit, Graves' orbitopathy	RPF[124,129-131,142-145]

Riedel's thyroiditis		RPF[132,133,146-148] MFF[127,128,147,149,150]

Chronic sclerosing sialadenitis[151]	Kuttner's tumour	

Panniculitis	Weber-Christian syndrome steatonecrosis, necrosing panniculitis	Systemic[110], RPF[135,152-155] Biliary cirrhosis[156], pancreas[157]

Sclerosing pancreatitis[158]	Primary inflammatory pancreatitis (P)[159], lymphoplasmacytic sclerosing P[7,160,161], autoimmune P[162], sclerosing pancreaticocholangitis[163], pancreatic pseudo-tumour[95]	RPF[5,97,139,161,164,165], cholangitis[95,160,164,166,167], systemic[83,168-171] gastric ulcer[172], lung[173,174] panniculitis[157], mesentery[175]

Sclerosing cholangitis		MFF[128], pancreas[92,166]

Bronchiolitis obliterans with organizing pneumonia	Cryptogenic organizing pneumonia, pulmonary hyalinizing granuloma.	Systemic[50,176], pancreas[173,174], liver[177], RPF[115,136,137], renal[46,136], mediastinum[178,179]

Benign pleural mesothelioma[180,181], calcifying pseudotumour[182,183]	Asbestos-related?	RPF[11,26-28,32,32] peritoneum[56,184,185] pleura[54,55,186]

RPF, retroperitoneal fibrosis; MFF, multifocal fibrosclerosis

In another patient, we observed the rare behaviour of idiopathic RPF behaving like a tumour, in which the inflammatory mass was invading both the kidney and liver and presented as a progressive cholangitis. This case was one of a series of patients seen in our gastroenterological department, who had sclerosing pancreatitis and chronic inflammation associated with immunoglobulin (Ig)G4-expressing plasma cells [3,4]. In biopsies from both these cases, we found typical fibrosis with lymphoplasmacytic inflammation and IgG4-bearing plasma cells [5].

Sclerosing (autoimmune) pancreatitis is a unique form of pancreatitis, characterized by hypergammaglobulinaemia and a lymphoplasmacytic inflammation of the pancreas that responds to glucocorticoid treatment. It has several synonyms (Table 2). A Japanese group investigating this condition [6] found that the sera of their patients had a polyclonal band in the rapidly migrating fraction of gammaglobulins, which was caused by a high concentration of the IgG4 gammaglobulin fraction. They also reported that serum concentrations of IgG4 were significantly and specifically raised in patients with sclerosing pancreatitis, and were closely associated with disease activity [6].

The same Japanese group found that of the 22 patients with sclerosing pancreatitis they studied, three also had concomitant hydronephrosis caused by a periureteric mass, diagnosed as RPF (Ormond's disease). Histological examination of the periureteric tissue showed abundant infiltration of IgG4-bearing plasma cells. Treatment with corticosteroids lowered serum concentrations of IgG4, and the authors proposed that IgG4 might have a pathological role in a systemic fibrosing process that includes pancreatic and retroperitoneal lesions [5].

Kamisawa et al have also published several reports emphasising the systemic nature of this process. Patients with autoimmune pancreatitis may also have involvement of lymph nodes and salivary glands as well as local tissues [7,8], in a pattern suggestive of multifocal fibrosclerosis [9,10].

We describe our index case, and report on the histological features of 11 other patients seen at our hospital with idiopathic RPF, for whom we have now been able to review the histology and examine sections for evidence of IgG4-expressing plasma cells. We also list the synonyms and disease associations that we believe are all part of the same disease process.

## Methods

### Histological examination

We report on the histological features of 16 biopsy specimens from 12 patients seen at the Middlesex hospital with a primary diagnosis of RPF, for whom we have now been able to review the histology and examine sections for evidence of IgG4-expressing plasma cells. Only archival paraffin wax-embedded material was used for this study. All the biopsies were taken for diagnostic reasons with the informed consent of patients.

All the samples were routinely fixed in formalin and embedded in paraffin wax. Dewaxed sections (μm) were stained with haematoxylin and eosin. Immunohistochemical analysis was performed using antibodies (all Dako UK Ltd, Cambridgeshire, UK) against CD3, CD4, CD8, CD20, and CD138, and the kappa and lambda light chains of immunoglobulins, by the standard streptavidin-biotin-peroxidase method. For IgG4, a mouse human IgG4 monoclonal antibody was used (1:100 dilution, MC011; The Binding Site, Birmingham, UK). Negative controls were created by substituting the primary antibody with similarly diluted non-immunized mouse serum.

We examined a wide variety of other inflammatory conditions and fibrotic diseases as organ-specific controls, which included liver (autoimmune hepatitis, chronic hepatitis C), pancreas (chronic and alcoholic pancreatitis), salivary gland (chronic sialadenitis), gallbladder (chronic cholecystitis), kidney (tubulointerstitial nephritis), duodenum (chronic duodenitis), colon (non-specific colitis), and other fibrotic processes (Dupuytren's, keloid scars). Because an association between RPF and asbestos exposure has been reported [11], we also included cases of malignant mesotheliomas. In addition, specimens from cases of "benign pleural plaques" were stained as controls.

Positive controls comprised tissue obtained from pancreas, salivary gland and liver from patients seen in our gastroenterological department who had sclerosing pancreatitis and chronic inflammation associated with IgG4-expressing plasma cells, details of which have been reported previously [3,4].

The degree of inflammation was assessed as severe [>100 inflammatory cells per high power field (), moderate (30–100 cells/HPF) and mild (30–10 cells/HPF). The presence of lymphoid-follicle formation, eosinophilic infiltration, venulitis and obliterative arteritis (on elastic van Gieson-stained sections) was also recorded. The number of IgG4-positive cells was estimated by counting 10 HPF, and scoring as follows: 0 (no IgG4 plasma cells/HPF), 1 (≤ 20 cells/HPF), 2 (20–50 cells/HPF) or 3 (≥ 50 cells/HPF) [12]. Fibrosis was assessed as - (no fibrosis), + (mild), ++ (moderate) or +++ (severe).

### Literature review

We reviewed the published literature for disease associations with idiopathic, systemic fibrosing conditions using the following synonyms: pseudotumour, myofibroblastic tumour, plasma cell granuloma, systemic fibrosis, xanthofibrogranulomatosis and multifocal fibrosclerosis. We also looked for reports associating these conditions with organ-specific conditions, particularly sclerosing pancreatitis (see Table 2 for other synonyms), RPF, Reidel's thyroiditis, panniculitis, Weber-Christian syndrome and retro-orbital tumour, and with a range of autoimmune diseases (Table 3).

**Table 3:** Rare associations with IgG4-related conditions (Case reports)

**Head and neck**	
Submandibular gland fibrosis	Pancreas[95], cholangitis[187,188], pseudotumour[189]
Parotid involvement	pancreas[171]
Sjögren's syndrome	Pancreas[171,190], RPF[164], BOOP[191]
Maculopathy	MFF[126]
Uveitis	RPF[192]
Conjunctiva	Pseudotumour[86]
Hashimoto's disease, Graves' disease	RPF[193]

**Cardiovascular**	

Constrictive pericarditis	RPF[194]
Heart valves	RPF[20,22]
Mediastinal fibrosis	RPF[194-196]
Chronic periaortitis/aneurysm	RPF[197-205]
Vasculitis	RPF[125,206,207]
Occlusive phlebitis	MFF[149]
Intermittent claudication	RPF[208]

**Rheumatology**	

Rheumatoid arthritis	BOOP[176], RPF[209]
Polymyalgia rheumatica	BOOP[210]
Ankylosing spondylitis	RPF[211]
Periarticular fibrosis	RPF[212]
Skeletal hyperostosis	RPF[213]
Dermatomyositis	Pseudotumour[88], BOOP[214]
Polymyositis	BOOP[191,215]
Scleroderma	RPF[216]

**Other autoimmune conditions**	

Systemic lupus erythematosus	RPF[217]
Idiopathic thrombocytopenic purpura	RPF[218,219]
Haemolytic anaemia	Lung[220], cholangitis[221]

**Central nervous system**	

Brain	PCG[107], xanthogranuloma[118,222]
Sella	MFF[124,126]
Spinal cord compression	RPF[223]

**Gastrointestinal**	

Primary biliary cirrhosis	RPF[224], BOOP[176]
Mesentery	RPF[112,152,175,225], pseudotumour[102,153,155]
Bladder/pelvic mass	RPF[49,208,226,227]
Duodenal obstruction	RPF[228,229]
Portal hypertension	RPF[230]

**Others**	

Familial multifocal fibrosclerosis Familial RPF	MFF[43] RPF[42]
Proliferative crescentic glomerulonephritis, membranous glomerulonephritis	RPF[231] RPF[232]
Interstitial nephritis	Pancreas[233]
Testicular involvement	RPF[126,234]

MFF, multifocal fibrosclerosis; RPF, retroperitoneal fibrosis; BOOP, bronchiolitis obliterans with organizing pneumonia; PCG, plasma-cell granuloma.

Many of these reports are case reports. We have not attempted to list every report but rather to draw up a comprehensive list of all possible associations with at least one citation.

## Results

### Index case

A 39-year-old Moroccan steel fitter had lived in the UK for 31 years. He presented in May 2003 with abdominal pain, weight loss, sweating, generalized lymphadenopathy, and severe anaemia and thrombocytosis. Investigations showed haemoglobin of 6.7 g/dL (normal range 13.0–17.0 g/dL), platelets 1031 × 10^9^/L (150–400 × 10^9^/L), erythrocyte sedimentation rate (ESR) 120 mm/h (0–15 mm/h), IgG 21.4 g/L (8.0–18.0 g/L), IgA 2.9 g/L (0.9–4.5 g/L), IgM 0.6 g/L (0.6–2.8 g/L) and C-reactive protein (CRP) 289 mg/L (normal 0–5 mg/L). Biochemistry was normal. Chest X-ray showed apical pleural thickening. An intravenous urogram (IVU) showed left hydronephrosis; an ultrasound scan (USS) confirmed this, and showed dilatation of the bile and pancreatic ducts and the gallbladder. Computed tomography (CT) showed multifocal upper zone and patchy nodular airspace shadowing, with right posterior pleural thickening and subcarinal lymphadenopathy. Biopsies of pleural, ileal and colonic tissue, bone marrow, and left inguinal lymph node were all either normal or showed reactive change. Cultures of blood, urine and sputum were all negative. The patient was also negative for human immunodeficiency virus types 1 and 2, hepatitis B and C, human T-lymphotropic virus, toxoplasmosis and treponemal disease. Mantoux test was weakly positive, and although bronchoalveolar lavage was smear-negative, there was one positive culture for a mycobacterium after 3 weeks.

The patient was commenced on quadruple therapy for tuberculosis (TB) in July 2003, for an organism that was strongly isoniazid-resistant. The patient presented again in November with fatigue, weight loss and fever, which was thought to be medication-related. He was given prednisolone 20 mg for 6 weeks of, taken off all TB therapy, and after 2 months was feeling so well that he was told his TB had been cured.

He re-presented 3 months later (March 2004) with a 6-week history of night sweats, low-grade fever, lethargy, weight loss, left pleuritic chest pain and axillary lymphadenopathy. Blood tests again showed microcytic anaemia, and the same acute phase response. Antinuclear antibody (ANA), antineutrophil cytoplasmic antibodies, mitochondrial antibodies and smooth-muscle antibodies were all negative. CT showed many of the previous changes but with a right hydronephrosis that was confirmed on IVU (left hydronephrosis was no longer present). A repeat bone-marrow biopsy, inguinal lymph node and transjugular liver biopsies, endoscopy, colonoscopy, peritoneal fluid aspiration and bone scan were all either normal or non-specific. The patient was treated with quintuple anti-TB therapy in order to cover his original mycobacterium infection as well as an infection with *Mycobacterium fortuitum *that had been cultured from sputum on this occasion. He was also treated with steroids, initially 60 mg of prednisolone, tapered down to 20 mg over 3 months.

The patient was readmitted 6 months later with generalized abdominal pain and vomiting. He had stopped his steroids 11 days earlier because of side effects. Investigations showed only a marked inflammatory response. CT scan revealed no hydronephrosis, but now showed dilated, thick-walled small bowel in the left iliac fossa. During an exploratory laparotomy (October 2004), an inflamed omental mass adherent to the small-bowel mesentery was removed. Histology was reported as showing only some fibrosis with a mild chronic inflammatory cell infiltrate of lymphocytes and plasma cells. The patient was recommenced on steroids and quadruple anti-TB therapy.

He remained well for some months, but then presented (March 2005) with a 6-week history of pain in the right upper quadrant of the abdomen. His blood tests showed the same inflammatory response and a USS showed prominent intrahepatic ducts only. The histology of the omental mass was reviewed and showed a large number of IgG4-positve plasma cells throughout the fibroinflammatory reaction (Table 4). The patient's serum IgG concentration was 21.4 g/l and IgG4 2.4 (normal range 0–1.3 g/l). A diagnosis of hyper-IgG4 disease was made, all microbial therapy was stopped, and the patient was treated with prednisolone 20 mg/day with immediate improvement in his illness. He remains on azathioprine and 5 mg prednisolone.

**Table 4:** Histopathology

Patient no., sex, age	Organ	Fibrosis*	Lymphoplasmacytic infiltrate†	Lymphoid follicles	Venulitis	Eosinophilic component	IgG4-positive plasma cells‡
(1) Male, 52	Kidney	+++	+++	Yes	No	No	3
	Liver	++	+	No	No	Yes	0
(2) Male, 73	Retroperitoneum	+++	+++	Yes	Yes	Yes	3
(3) Male, 39	Omentum	+	++	Yes	No	No	3
(4) Male, 53	Retroperitoneum	+++	+++	Yes	Yes	Yes	3
(5) Male, 45	Periureter	+++	+++	Yes	Yes	No	2
(6) Female, 58	Retroperitoneum	+++	+++	No	Yes	No	3
(7) Female, 55	Para-aortic tissue	+++	+++	Yes	Yes	No	3
(8) Male, 47	Periureter tissue	+++	+	Yes	Yes	No	2
(9) Male, 48	Retroperitoneum	+++	++	No	No	No	2
(10) Male, 73	Retroperitoneum	+++	+++	Yes	Yes	Yes	1
(11) Female, 56	Retroperitoneum	+++	++	Yes	No	No	1
(12) Male, 53	Liver	++	+	No	No	No	1
	Colon	++	+	No	No	No	0

*Degree of interstitial fibrosis: +, mild; ++, moderate; +++, severe fibrosis.

†Degree of inflammation: +++ (severe), >100 inflammatory cells/HPF; ++ (moderate), 30–100 cells/HPF; + (mild), 10–30 cells/HPF

‡IgG4-positive plasma cells: 0, no IgG4 plasma cells/high power field (HPF); 1, ≤ 20 cells/HPF), 2, 20–50 cells/HPF; 3, ≥ 50 cells/HPF (criteria of Aoki et al [12]).

### Pathology of IgG4 disease

We reviewed the histology and examined sections for evidence of IgG4-expressing plasma cells of 16 biopsies from 12 patients with a clinical diagnosis of idiopathic RPF seen at our hospital in the past 10 years. Biopsies comprised nine samples of retroperitoneal tissue, two of liver, and one each from kidney, colon and omentum. In addition, there were two further samples of retroperitoneal tissue taken from two patients after steroid therapy.

**Figure 1:**
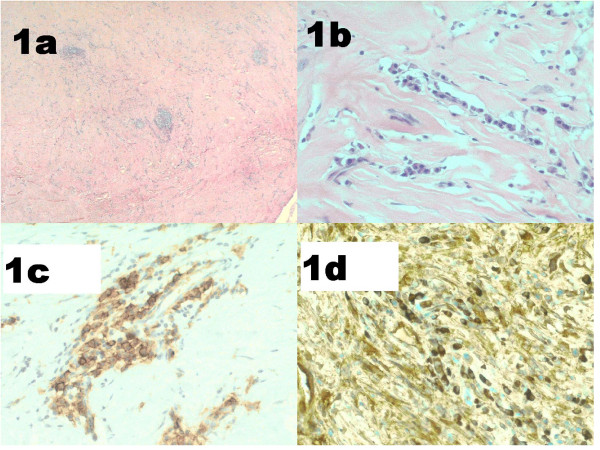
**Mesenteric mass**. Patient 3 (index case): (**a, b**) fibrosis, lymphoid aggregates and plasma cells (haematoxylin and eosin (a) × 100, (b) ×400); (**c**) CD138-positive plasma cells; (**d**) IgG4-positive plasma cells.

**Figure 2:**
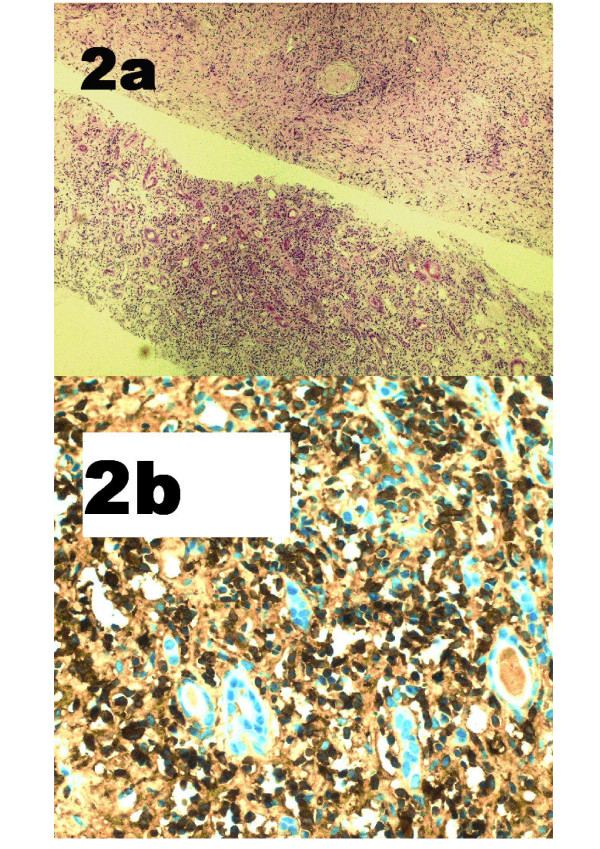
**Renal biopsy**. Patient 1, showing renal involvement. (**a**) Cores of renal tissue with a conspicuous component of inter- and intra-tubular inflammatory cells (haematoxylin and eosin ×100); (**b**) IgG4-positive plasma cells.

**Figure 3:**
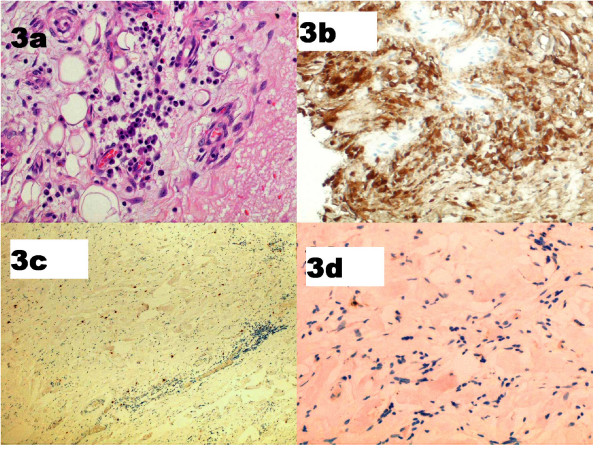
**Retroperitoneal tissue**. Patient 7. Pre-glucocorticoid treatment: (**a**) fibrosis and plasma cells (haematoxylin and eosin ×400); (**b**) IgG4-positive plasma cells. Post-glucocorticoid treatment: (**c**) scattered CD138-positive plasma cells; (**d**) IgG4 staining reveals no positive cells.

**Figure 4:**
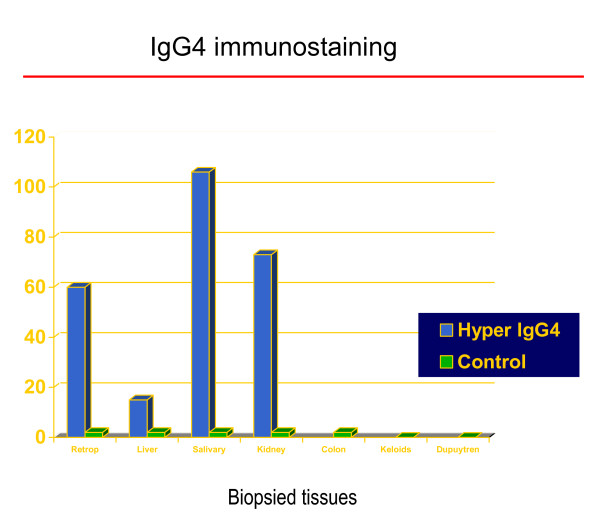
**Histopathology: IgG4 immunostaining (cases versus controls)**. Vertical axis (0–120) shows number of IgG4-positive plasma cells/high power field (see Methods for details). Histological data from salivary gland is positive control, (from previously reported study [3,4]).

**Figure 5:**
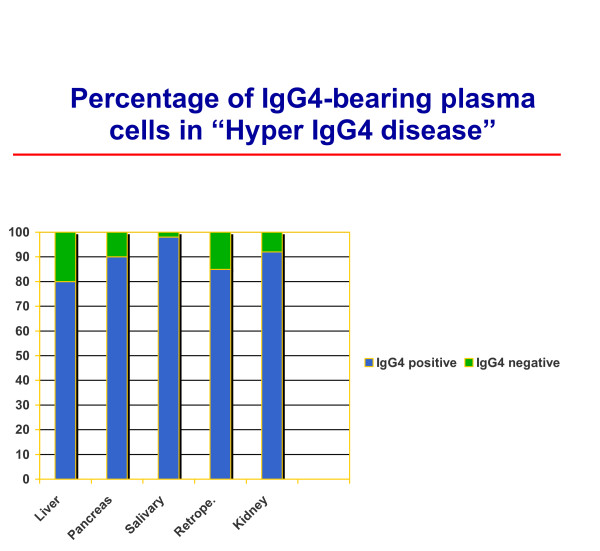
Percentage of IgG4-bearing plasma cells.

The results are shown in Table 4. All patients showed, to varying degrees, fibrosis, intense inflammatory cell infiltration with lymphocytes, plasma cells, scattered neutrophils and in some cases eosinophilic aggregates. The majority of lymphocytes expressed CD8 and CD4 positivity (T-cell markers), with B cells present to a lesser degree and with scattered B-cell-rich small lymphoid follicles. In addition, we saw vasculitis affecting small veins, and evidence of obliterative arteritis (Figures 1, 2, 3). In all cases, there was a significant increase in IgG4-positive plasma cells compared with controls (Figure 4). Even when few plasma cells were seen (patients 10–12) the majority expressed IgG4 (Figure 5). In two cases, biopsies before and after steroid treatment were available; only scattered plasma cells were seen after treatment, none of which expressed IgG4 (Fig 3). In all of our patients, the kappa and lambda light chains showed a polytypic pattern. We used PCR to look for evidence of oligoclonal infiltrates for IgH in one case (patient 1), as has been reported from Japan [13], but found none. None of the wide range of control sections showed significantly increased numbers of IgG4-positive cells.

We also examined biopsy specimens from liver (two patients), large bowel (one) and renal tissue (one). In all of them, we found mild fibrosis and a small increase in lymphoplasmacytic inflammatory cell infiltrate, although IgG4-positive plasma cells were not seen in one liver biopsy, nor in the colon biopsy (Table 4).

In summary, raised numbers of IgG4-positive plasma cells are seen in both peritoneal and non-peritoneal tissues in patients with hyper-IgG4 disease. The degree of IgG4-positive staining did not necessarily correlate with elevated serum IgG. Our clinical impression was that the number of IgG4-positive plasma cells was associated with disease activity.

## Discussion

RPF is probably the most common example of the conditions associated with raised serum IgG4 and an IgG4-positive plasma cell infiltrate, which we call hyper-IgG4 diseases. The other most frequently recognised associations are sclerosing pancreatitis, Reidel's thyroiditis, retro-orbital tumour, panniculitis, and sclerosing cholangitis (see Table 2 for synonyms).

It is known that RPF can be associated with a similar fibrotic process in other organs, particularly adjacent tissues such as the mediastinum and pleura. Patients with RPF can have systemic symptoms and be febrile. We used RPF and our knowledge of its aetiology and pathogenesis to review the potential mechanisms of IgG4 disease.

### Retroperitoneal disease

Idiopathic RPF generally presents in a non-specific manner with malaise, fatigue, fever and weight loss [11,14-16]. Pain in the back (lumbosacral) region, flank or lower abdomen is most common. The pathognomonic feature of RPF is a thick retroperitoneal fibrotic mass covering the abdominal aorta and compressing the ureters. The process of fibrosis can result in obstruction of the ureters and renal failure, or signs and symptoms may be related to the encasement or entrapment of other structures by the inflammatory mass, such as hydrocoeles or unilateral leg oedema. With renal involvement, severe hypertension is common.

The disease presents typically between the ages of 50 and 70 years, and men are affected 2–3 times more commonly than women [14]. The inflammatory nature of idiopathic RPF is demonstrated by elevated ESR and CRP, reduced haemoglobin and elevated gammaglobulins at presentation. An autoimmune nature is suggested by the finding of ANA in 10–20% of cases, an occasional association with different autoimmune diseases and good response to glucocorticosteroids. In one series of RPF associated with periaortitis, 10 of 16 patients were ANA-positive [17].

Although there are no randomised studies of treatment for RPF, patients who present during the acute inflammatory phase respond quickly to glucocorticosteroids. Patients with late presentation and dense fibrosis will not respond to medical treatment and surgery is often necessary[11].

#### Causes and associations

RPF has many causes, although in about 70% of cases the cause is unknown (idiopathic). The rest (secondary) develop as a result of malignant disease, radiation therapy, abdominal surgery, pancreatitis, retroperitoneal haemorrhage, urine extravasation, and infections. Carcinoid syndrome can cause systemic fibrosis [18], and RPF has been described without metastatic tumour [19].

##### Drugs

RPF has also been associated with the use of several drugs, but most commonly the ergotamine derivates, especially methysergide [20] and, more recently, those used as dopamine agonists to treat Parkinson's disease, such as pergolide and cabergoline [21].

From an aetiological perspective, drugs associated with RPF are important as they also cause fibrosis in various other sites. Systemic fibrosis is a prominent feature of methysergide use, with involvement of heart and lungs, particularly heart valves [20,22]. A similar distribution of pathology has been reported with the dopamine agonists pergolide and cabergoline [23-25].

Of pathogenetic importance is the observation that withdrawal of these offending drugs usually results in a rapid improvement in symptoms, reduction in inflammatory markers and regression of pathology [20]. Drug-induced cases are not associated with ANA [16].

##### Asbestos

Recently, asbestos exposure has been proposed as a causal factor for both pleural and retroperitoneal fibrosis [11,26-28]. Asbestos fibres also cause interstitial lung fibrosis (asbestosis), pleural fibrosis, pleural plaques, lung cancer, and pleural and peritoneal mesothelioma [29]. Patients exposed to asbestos, and particularly those with asbestosis, have an increased incidence of ANA and other autoantibodies, and raised levels of IgG and IgA. There is also a correlation between the degree of immunological abnormality and the likelihood of progression [30,31].

A case-control study in Finland of 43 patients with RPF (86% of eligible cases) found that the age-standardised incidence of RPF was 0.10 per 100000 person-years [11]. For every patient, five population-based matched controls were selected. There was a strong association with asbestos exposure. The odds ratio (OR) was 5.5 for <10 fibre-years of asbestos exposure and 8.8 for ≤ 10 fibre-years. Other risk factors were previous use of ergot derivates (OR = 9.92), abdominal aortic aneurysm (OR = 6.73) and smoking for >20 pack-years (OR = 4.73) [11].

We also saw pleural calcification in three of our patients with RPF, in whom it was not present at the presentation of the illness. One of those cases is included in our archival review (patient 5, Table 4). This raises the question of whether some of the pleural pathology associated with IgG4 disease can heal with calcification as well as fibrosis [32].

##### Aortitis

The relationship of aortic aneurysms with an inflamed and thickened aortic wall, extensive periaortal inflammation and RPF is well known [33]. As the inflammatory aneurysms of the abdominal aorta differ from RPF only in the diameter of the inflamed aorta, it has been suggested that both syndromes represent only variations of the same disease, which has been named 'chronic periaortitis' [34]. RPF has been suggested as the most severe form of chronic periaortitis [35,36]. Vasculitis of the vasa vasorum was thought to be a factor in the aneurysmal dilatation [34,37].

Several observations suggest an immunological pathogenesis for this periaortitis. As well as the periaortal tissue, the media and adventitia of the aorta are infiltrated by polyclonal B lymphocytes, activated CD4-positive T lymphocytes, and plasma cells [35]. In 85% of patients, the necrotic media contains deposits of IgG antibodies in close proximity to extracellular ceroid, and the serum often contains antibodies against ceroid and oxidised low-density lipoprotein [38].

Although it was initially proposed that the aortitis was caused by an autoimmune response to the components of atherosclerotic plaques [38] with antibodies to atheroma, more recent opinion has suggested that in view of the accompanying systemic symptoms and acute phase response, the aortitis is a primary autoimmune disease [17,36,39,40].

##### Autoimmune and other rare associations

While there is no one predominant association, a large range of autoimmune diseases has been described in association with RPF (Table 3), and 10–20% of cases will have ANA or other autoantibodies.

##### Paediatric

RPF in the paediatric population is rare with, only 23 cases reported in the English-language literature by 2003 [41]. A family has been reported in which two children had idiopathic retroperitoneal fibrosis, and they and their father had clinical and laboratory evidence of systemic immunological diseases [42].

##### Familial

Two brothers from a first-cousin consanguineous marriage were reported, who had different combinations of RPF, mediastinal fibrosis, sclerosing cholangitis, Riedel sclerosing thyroiditis, and pseudotumour of the orbit. One of the brothers had fibrotic contracture of the fingers [43]. Other reports are of RPF in three siblings with sickle-cell trait [44], and mediastinal and retroperitoneal fibrosis in two sisters with seronegative spondyloarthropathy [45].

#### RPF invading adjacent organs

Rarely, the inflammatory tissue seen in RPF can invade the renal parenchyma [46-48] and it can occasionally cause a tumour-like mass away from the retroperitoneum [49]. There are four isolated surgical case reports in the literature, with two being described as idiopathic RPF, one as systemic hyalinizing granuloma and the other as xanthofibrogranuloma. We have seen one case in which the IgG4-positive inflammatory mass (Figure 2) invaded the kidney and liver (patient 1, Table 4), and another in which it caused a large tumour like mass in the left iliac fossa (patient 2). Both cases responded promptly to steroids.

#### Summary

Although all our archival cases of idiopathic RPF have shown evidence of IgG4 involvement, it remains to be seen whether IgG4 plays any role in other types of RPF such as periaortitis, drug-induced and asbestos-related RPF. We certainly found it (in retroperitoneal tissue) in one of our patients who developed pleural calcification.

### Lung disease

Part of the spectrum of disease in patients with hyper-IgG4 disease is parenchymal lung pathology, often described as either bronchiolitis obliterans with organizing pneumonia or cryptogenic organizing pneumonia [50]. This is seen typically as migratory pulmonary infiltrates, and is steroid-responsive. Nevertheless, there are at present no studies that have looked for evidence of IgG4 plasma cells in these conditions [51]. Other conditions associated with migratory pulmonary infiltrates that may be related, but not yet investigated, include pulmonary eosinophilia, hypersensitivity to drugs, parasitic infection, allergic bronchopulmonary aspergillosis and Churg-Strauss vasculitis [52]. Pleural thickening, with and without calcification, is well described [32,32,53]. There are also reports of calcifying pseudo-tumour of the pleura [54,55] and peritoneum [56].

### Differential diagnosis of febrile illness

Fever of unknown origin (FUO) in an immunocompetent patient is defined as an illness of more than 3 weeks' duration with fever higher than 38.3°C (101°F) on several occasions, and with an uncertain diagnosis after 1 week of investigation [57]. Infection accounts for about one-third of cases (25–32%) of FUO, followed by non-infectious inflammatory disease (21–31%) and neoplasm (12–17%) [58-62]. Although many patients with FUO will be found to have bacterial infection, rarer diagnoses will be considered with time and extensive investigation. Nevertheless, at the end of all investigations, 18–30% of patients will have no diagnosis, although the majority of this idiopathic group will improve spontaneously [60]. We propose that hyper-IgG4 disease can be added to this list of systemic inflammatory diseases.

### Pathogenesis

#### Conditions associated with elevated IgG4

IgG4 is the rarest of the IgG subclasses to be expressed, accounting for only 3–6% of total IgG in normal serum. It has a low affinity for target antigen. It is unique among the IgG subclasses, as it is unable to bind Clq complement and cannot activate the classic complement pathway [63]. IgG4 is a T-helper 2 (Th2)-dependent isotype. Interleukin (IL)-4 directs naive human B cells to switch to IgG4 and IgE production [64]; IL-10 and IL-13 are the other T-cell-derived cytokines that activate this differentiation.

High serum IgG4 concentrations are found in a limited number of other conditions, including atopic dermatitis [65], some parasitic diseases (helminthic diseases) [66], and pemphigus vulgaris (PV) and pemphigus foliaceus (PF)[67,68].

In allergic individuals, a feature of successful immunotherapy is the induction of allergen-specific IgG4 antibodies, even with the persistence of allergen-specific IgE [69,70]. High levels of the immunosuppressive cytokine IL-10 are found both in patients chronically infected with helminths and in those receiving allergen immunotherapy [71]. IL-10 also stimulates IgG4 production [72].

Th2 responses to allergens are suppressed by both CD4+ CD25+ T regulatory cells (Tregs) and IL-10-producing Tregs [73], and suppression by these subsets is decreased in allergic individuals. In animal models, Tregs can be induced by high- or low-dose inhaled antigen, and prior induction of Tregs prevents subsequent development of allergen sensitization and airway inflammation in inhaled challenge models. Allergen-injection immunotherapy may induce IL-10 Tregs, leading to both suppression of Th2 responses and a switch from IgE to IgG4 antibody production [73].

##### Helminths

Asymptomatic infections with helminth parasites are correlated with high levels of IgG4, and parasite-specific IgG4 antibodies can inhibit IgE-mediated degranulation of mast cells [66]. In most helminth infections, the Th2 response leads to rapid parasite expulsion or sequestration. During murine *Schistosoma mansoni *infection, however, parasites persist and the chronic Th2 response can induce severe pathological fibrosis in the gut and liver [74]. Evidence both from these mouse models and from human studies suggests that this progression to severe disease with intense fibrosis occurs in only a minority of cases, and is a consequence of the strongly profibrotic type 2 cytokine response mediated by IL-4 and IL-13 [74].

##### Pemphigus and pemphigoid

PV and PF are autoimmune skin diseases caused by autoantibodies against desmoglein 3 and desmoglein 1 respectively. These antibodies are predominantly of the IgG4 subclass [67,68]. Desmogleins are keratinocyte transmembrane proteins localized in the desmosome, and are members of the adhesion molecule family of cadherins. The interaction of antidesmoglein antibodies with their target antigens leads to loss of cell adhesion (acantholysis) and the formation of intraepithelial blisters of the skin and mucous membranes. The antibodies predominantly, but not exclusively, bind to the extracellular domains of the proteins and exert their pathogenic effect not through inhibition of the proteins' adhesive properties, but through an indirect pathway involving cellular signalling pathways, resulting in destabilization of desmoglein 1-based adhesive sites and desmosomes [75].

Bullous pemphigoid (BP) is an autoimmune blistering disease associated with autoantibodies against the hemidesmosomal glycoprotein BP180. The noncollagenous (NC)16A domain of BP180 has major antigenic sites recognized by BP sera. IgG4 and IgE are the major immunoglobulins that preferentially react with two distinct epitopes within this domain. Levels of these autoantibodies correlated with disease activity in BP [76].

##### Autoimmune pancreatitis

In sclerosing pancreatitis, evidence had been reported to suggest an immunological pathogenesis even before the description of a role for IgG4. In one study of 17 patients, ANA and antilactoferrin antibodies were detected in 76%, carbonic anhydrase II antibody in 59%, rheumatoid factor in 29% and anti-smooth-muscle antibody in 18% of patients. CD8+ and CD4+ cells were significantly increased in peripheral blood. CD4+ cells produced increased levels of interferon gamma compared with controls, but IL-4 was not increased [77]. However, in another Japanese study, which divided patients with sclerosing pancreatitis into a seronegative and seropositive group, found no differences in presentation, pathology or natural history between the groups [78].

##### HLA associations with IgG4-associated diseases

• PV was consistently found to be associated with DR4 and DR14 and, more precisely, with DRB1*0402 and DRB1*1401 subtypes [79]. Susceptibility to PF has been correlated with the presence of DR4, DR14 and DR1 alleles; however, in contrast to PV, no single DR4 or DR14 allele was shown to be associated with the disease.

• In an immunogenetic study of patients with autoimmune pancreatitis, a significant increase in DR4 was found. It was concluded that the DRB1*0405-DQB1*0401 haplotype was associated with autoimmune pancreatitis in the Japanese population [80].

• Aortoarteritis is a chronic inflammatory disease, mainly affecting the aorta and its major branches. In a Chinese Han population, the DRB1*04 and DRB1*07 alleles were significantly associated with aortoarteritis. Furthermore, there was no significant difference in the frequency of the DRB1*0405 subtype between the patient and control groups [81].

• In China, the HLA-DRB5*0101 allele and the IL-13 promoter A/A genotype were both found to be elevated in schistosomal hepatic fibrosis, although the two genes are located on different chromosomes. Subjects with both genotypes had odds ratios much higher (OR = 24.5) than the sum of the ratios for each individual genotype. The study strongly suggested that a pathogenic Th2 response directly influenced the prognosis of post-schistosomal liver fibrosis[82].

## Conclusion

We believe that hyper-IgG4 disease is an important condition to recognise as the diagnosis can be verified by histology and simple blood tests, and the outcome with treatment during the acute phase is very good. A raised IgG concentration in the absence of hypergammaglobulinaemia is typical, but ideally IgG4 should also be measured. Even when pathology appears localised, as with RPF, constitutional symptoms can be present with evidence of an acute phase response. Rarely a systemic form of the disease will present with severe constitutional symptoms, and hyper-IgG4 disease can be added to list of unusual causes of FUO. Although the role and relevance of IgG4 is unknown, the dense fibrosis that accompanies healing would appear to be a typical example of Th2-mediated pathology.

## Competing interests

The author(s) declare that they have no competing interests.

## Authors' contributions

GH Neild was involved with clinical management of the patients and was responsible for the writing of the paper. Manuel Rodriguez-Justo reviewed and examined all the histological material and helped to write the paper. Catherine Wall and John O Connolly were both involved with the clinical management of the index case and several of the patients with RPF, and helped to write the paper.

## Pre-publication history

The pre-publication history for this paper can be accessed here:


